# Secretome Analysis of *Macrophomina phaseolina* Identifies an Array of Putative Virulence Factors Responsible for Charcoal Rot Disease in Plants

**DOI:** 10.3389/fmicb.2022.847832

**Published:** 2022-04-05

**Authors:** Nilanjan Sinha, Sourav Kumar Patra, Sanjay Ghosh

**Affiliations:** Department of Biochemistry, University of Calcutta, Kolkata, India

**Keywords:** *Macrophomina phaseolina*, charcoal rot disease, solid-state culture, secretome analysis, putative virulence factors, LC-MS analysis, cell wall degrading enzymes, proteases

## Abstract

*Macrophomina phaseolina* is a global devastating necrotrophic fungal pathogen. It causes charcoal rot disease in more than 500 host plants including major food crops, pulse crops, fiber crops, and oil crops. Despite having the whole-genome sequence of *M. phaseolina*, understanding the *M. phaseolina* genome-based plant–pathogen interactions is limited in the absence of direct experimental proof of secretion. Thus, it is essential to understand the host–microbe interaction and the disease pathogenesis, which can ensure global agricultural crop production and security. An *in silico*–predicted secretome of *M. phaseolina* is unable to represent the actual secretome. We could identify 117 proteins present in the secretome of *M. phaseolina* using liquid chromatography–electrospray ionization–tandem mass spectrometry. Data are available *via* ProteomeXchange with identifier PXD032749. An array of putative virulence factors of *M. phaseolina* were identified in the present study using solid-state culture. Similar virulence factors have been reported in other plant pathogenic fungi also. Among the secretory fungal proteins with positive economic impacts, lignocellulolytic enzymes are of prime importance. Further, we validated our results by detecting the cell wall–degrading enzymes xylanase, endoglucanase, and amylase in the secretome of *M. phaseolina*. The present study may provide a better understanding about the necrotrophic fungi *M. phaseolina*, which modulate the host plant defense barriers using secretory proteins.

## Introduction

*Macrophomina phaseolina* is a global devastating necrotrophic fungal pathogen that causes charcoal rot disease in more than 500 host plants (Wyllie, 1998) including major food crops (Su et al., 2001), pulse crops (Raguchander et al., 1993; Mayek Pérez et al., 2001), fiber crops (De et al., 1992; Aly et al., 2007), and oil crops. Other diseases caused by this pathogen include color rot, root rot, and damping off; stem root and seedling blight in many economically important plants (Kishore Babu et al., 2007). *M. phaseolina* belongs to the Botryosphaeriaceae family (Crous et al., 2006). The occurrence of sclerotia of *M. phaseolina* in plant debris allows the fungus to live in soil, even in the absence of a host for 2 or more years, depending on soil conditions. Pathogenicity of *M. phaseolina* is optimal at 28–35°C, and host water stress is another principal factor favoring development of the disease (Dhingra and Sinclair, 1973). Emergence of *M. phaseolina* is now a serious concern for the cultivation of soybean under climate change scenario worldwide (Vibha, 2016). In India, this disease became a serious threat due to altered weather condition particularly because of the longer drought spells during crop growth period. Subsequently, the disease started to appear irregularly in some areas, which caused great amount of yield losses (Gupta et al., 2012).

In the last decade, proteomic analysis of secretory proteins of phytopathogenic fungus has been used increasingly to study the plant–pathogen interactions (Gonzalez-Fernandez and Jorrın-Novo, 2012). Proteomic tools such as gel-based or gel-free liquid chromatography–based mass spectrometry (LC-MS/MS) permit identification of key proteins of the secretome during host–microbe interactions. Enzymes secreted by certain fungi hold the key to its pathogenesis. Secretome study of *Trichoderma harzianum* shows 51.36% presence of glycoside hydrolases (GHs), and the secretome of this filamentous fungus is rich in hydrolytic enzymes (Do Vale et al., 2012). Carbohydrate-degrading enzymes and proteases were found in secretome analysis of *Diplodia corticola* (Fernandes et al., 2014). However, very little information is available about the secretome of fungi belonging to the Botryosphaeriaceae family due to the unavailability of whole-genome sequence of the fungus. However, this is not the case for *M. phaseolina*; its genome has already been sequenced. Whole-genome sequence of *M. phaseolina* shows the presence of 362 putative Cazymes including 219 GHs (Islam et al., 2012). Plant cell wall represents the first barrier to an invading pathogen, and *M. phaseolina* invades the plant defense by secreting a cocktail of enzymes (Le Marquer et al., 2019). Despite having the whole-genome sequence of *M. phaseolina*, understanding the *M. phaseolina* genome-based plant–pathogen interactions is not easy in the absence of direct experimental proof of secretion because an *in silico*–predicted secretome is unlikely to exactly represent the actual secretome. Thus, proteomic analysis of secretome will become instrumental in designing rational strategies for disease control. Not only that, but it is also essential to understand the host–microbe interaction and the disease pathogenesis, which can ensure global agricultural crop production and security. In our previous study, we observed induction of charcoal rot disease during *Corchorus capsularis* (jute) JRC 412–*M. phaseolina* (strain R9) interaction that resulted in elevated nitric oxide, reactive nitrogen species, and S-nitrosothiols production in infected tissues. To mimic the plant–pathogen interaction, *M. phaseolina* was grown on wheat bran in a solid-state culture, and secretome was collected. Interestingly, nitric oxide was produced in single leaf experiment in the presence of both *M. phaseolina* and xylanases obtained from fungal secretome. In fact, xylanase is known to play a vital role in presenting pathogen-associated molecular pattern (PAMP) which was also evident in our experimental model system (Sarkar et al., 2014). In the present study also, we used similar solid-state culture where *M. phaseolina* (strain R9) was grown on wheat bran bed to mimic the plant–pathogen interaction. We identified 117 proteins present in the secretome of *M. phaseolina* using LC–electrospray ionization–MS/MS (LC-ESI-MS/MS). An array of putative virulence factors of *M. phaseolina* were identified that have been reported to be involved in other plant pathogenic fungi also. Among the secretory fungal proteins with positive economic impacts, lignocellulolytic enzymes are of prime importance. Further, we validated our results by detecting the cell wall–degrading enzymes xylanase, endoglucanase, and amylase in the secretome of *M. phaseolina*. The present study may provide a better understanding about the necrotrophic fungi *M. phaseolina*, which modulate the host plant defense barriers using secretory proteins.

## Materials and Methods

### Microorganism and Maintenance of the Culture

A virulent isolate of *M. phaseolina* (strain R9) was collected from Sorbhog, Assam. A pure mycelial culture was generated through single sclerotia of this isolate, maintained in potato dextrose agar (PDA) media at 30°C. PDA slants were inoculated from the stock culture and maintained for several days at 30°C. Wheat bran was selected as the solid state for the experiments. Ten grams of wheat bran was taken in 100-mL conical flask, and it was sterilized. Spores of *M. phaseolina* were inoculated in wheat bran with the help of Mendel’s Mineral Salt Solution [for 1 L (NH_4_)_2_S0_4_ 1.4 g, K_2_HPO_4_ 2 g, CaCl_2_, 2H_2_O 0.4 g, MgSO_4_,7H_2_O 0.3 g, FeSO_4_,7H_2_O 5 mg, MnSO_4_,7H_2_O 1.6 mg, ZnSO_4_,7H_2_O 1.4 mg, CoCl_2_,6H_2_O 2 mg]. The wheat bran–containing conical flasks were then maintained at 30°C and 90% moisture content.

### Isolation of Secretory Protein

After specific day of incubation of conical flasks containing *M. phaseolina*, infected wheat bran was taken for protein isolation. An amount of 30–40 mL of sodium phosphate buffer of pH 7.0 was directly added to the conical flask containing infected wheat bran. The secretome was extracted twice from the infected wheat bran. For the first extraction, the conical flasks were placed in a shaker and rotated at 120 revolutions/min (rpm) for proper shaking at room temperature for 4 h. The buffer containing secretory proteins was extracted from wheat bran by compression using a glass rod from the conical flasks, and the decanted materials were filtered through Whatman filter paper (110 mm). For the second time extraction, an amount of 20–25 mL of 50 mM sodium phosphate buffer of pH 7.0 was again added directly to the wheat bran bed, and the conical flask was kept under shaking condition at 120 rpm for 2 more hours at room temperature. Extraction of the secretory proteins in 50 mM sodium phosphate buffer was done using similar procedure as described before. Concentration of the protein was measured after each extraction using Bradford reagent (Bradford, 1976). Secretory proteins collected after the first and second extractions were pooled, concentrated using 0–80% ammonium sulfate precipitation, and finally dialyzed to remove the salts from the concentrated proteins.

### One-Dimensional Gel Electrophoresis

*Macrophomina phaseolina* secretome was analyzed in sodium dodecyl sulfate–polyacrylamide gel electrophoresis (SDS-PAGE) to observe the protein bands along with the molecular weight markers. Secretome of 5-, 8-, 11-, and 14-day post-inoculated culture was loaded for separation on a 10% SDS-PAGE. Each lane contained 50 μg of protein sample.

### Identification of Proteins by LC-ESI-MS/MS

Extracted proteins from *M. phaseolina* secretome were first lyophilized and then solubilized in 100 mM ammonium bicarbonate. Tetra-fluoroethylene, dithiothreitol, and iodoacetamide were added sequentially to the solution. Then, trypsin was added and kept overnight at 37°C prior to LC-ESI-MS/MS. LC-ESI-MS/MS was done on Waters-Xevo-G2-X’S-QTof machine in ESI-positive mode. BEH C 18 column (Waters) was used in the instrument. The machine used MS^E^ scanning technique where MS and MS/MS took place simultaneously. Sodium formamide was used as primary standard, and leucine enkephalin was used as secondary standard. Ramp collision energy was set at 18–40 V. MS and MS/MS thresholds were set at 150 and 20 counts, respectively; 0.1% formic acid was added to the sample; 10 μL sample was injected finally for detection of the peptides. The run time was set 1 h. In the first 5 min (0–5 min), the column was being equilibrated. In the next 45 min (5–50 min), the peptides were eluted from the column with ACN gradient 0–90%. In the last 10 min (50–60 min), the column was being washed. The peptides detected by LC-MS/MS were matched with *M. phaseolina* MS6 database downloaded from UniProt in FASTA format. The software used for analysis is Progenesis Qip by Waters.

### Enzyme Assay

The activity of xylanase and endoglucanase was measured using dinitrosalicylic acid (DNS) assay method, which measures the total amount of reducing sugars. 3,5-DNS in the presence of reducing sugar forms 3-amino 5-nitrosalicylic acid, and this compound strongly absorbs light at 540 nm (Miller, 1959).

DNS reagent was prepared by dissolving 1 g of 3,5-DNA in 50 mL of water; 30 g of sodium potassium tartrate was added to the solution in small lots, and the solution turns milky yellow in color. Finally, 20 mL of 2(N) NaOH was added, and that turned the solution to transparent orange yellow color.

Xylanase activity was measured by using 1% birchwood xylan as substrate (Yang et al., 1995). Xylanase degrades xylan and forms xylose. The formation of xylose in 10 min at 30°C was measured by the DNS reagent. Xylose standard curve was plotted by taking known concentration of xylose and their corresponding optical density values at 540 nm (OD_540_). One unit of enzyme activity is defined as the amount of enzyme producing 1 μmol of xylose equivalents per minute under the given conditions.

Endoglucanase activity was determined in accordance with the International Union of Pure and Applied Chemistry recommendations, with a 1% solution of carboxymethyl (CM) cellulose as the substrate. Endoglucanase degrades CM cellulose and forms glucose. The formation of glucose in 10 min at 30°C was measured by the DNS reagent. Standard curve of glucose was obtained by taking known concentrations of glucose followed by addition of DNS reagents and their corresponding OD_540_ values (Dutta et al., 2008). One unit of enzyme activity is defined as the amount of enzyme producing 1 μmol of glucose equivalents per minute under the given conditions.

Amylase activity was measured by using starch as the substrate. Amylase hydrolyses starch into smaller carbohydrate molecules such as maltose. Iodine reacts with starch to form a starch–iodine complex. The color of the solution turns blue, and it strongly absorbs light at 620 nm (Cochran et al., 2008). Standard curve of starch was plotted by taking known concentrations of starch and their corresponding OD_620_ values in the presence of iodine. The starting concentration of starch was 250 μg. The degradation of starch occurred when it was incubated with the protein solution for 1 min. Iodine was added to the solution, and OD_620_ values were taken. The amount of starch degraded can be calculated with the OD_620_ values and the values from the starch standard curve. The activity of enzyme was calculated as the amount of starch degraded per minute per microgram of protein.

### Zymogram Analysis

Zymogram analysis is a great way to visualize the presence of active enzymes in the secretome. Zymogram of xylanase and endoglucanase was performed with a modified method described in Coral et al. (2002). Zymogram of xylanase and endoglucanase was done by casting the resolving gel with 0.1% birchwood xylan and 0.1% CM cellulose. Native-PAGE was done to keep the secretome proteins in active state. Native-PAGE gels (1.5-mm thickness) were prepared with 5% stacking gel and 8% resolving gel for both zymograms. The protein loading samples were prepared without SDS or β-mercaptoethanol to maintain the native state of the protein. Thirty micrograms of protein collected on days 5, 8, 11, and 14 after inoculation was loaded in each of the lanes. The Native-PAGE was run at 4°C. Electrophoresis was done using 30-mA steady current. After the dye front reached the bottom of the gel, it was further electrophoresed for 30 min. The gel was removed from the glass plates and placed in a glass dish. The gel was then stained with 0.1% Congo red and then destained with 1 M NaCl (Dutta et al., 2008). After destaining the gel, white regions can be seen in red color background. The gel pictures were taken on Gel DocTM XR+ BioRad imaging machine.

Zymogram of amylase was performed with a modified method described by Andrades and Contreras (2017). Zymogram of amylase was done by casting the resolving gel with 0.4% starch. A native polyacrylamide gel (1.5-mm thickness) was prepared with 5% stacking solution and 8% resolving solution. The protein loading samples were prepared without SDS or β-mercaptoethanol to maintain the native state of the protein. An amount of 10 μg of protein collected on days 5, 8, 11, and 14 after inoculation was loaded in each of the lanes. The gel was prepared and ran on the same day. The Native-PAGE was run at 4°C. Electrophoresis was done using 30-mA steady current. Electrophoresis was stopped after the dye front reached the bottom of the gel. The gel was removed from the glass plates and placed in a glass dish. The gel was stained with iodine solution. Chewed-up region of the gel can be seen as white in a blue/violet color background. The gel picture was taken on Gel DocTM XR+ BioRad imaging machine.

### Bioinformatics Tools Used in Analysis of *Macrophomina phaseolina* Secretome

Signal P is an online tool that predicts the presence of signal peptide in protein sequences. It is a very useful tool for secretome analysis. Proteins found in LC-ESI-MS/MS of *M. phaseolina* contained a unique UniProt ID. Sequences of specific proteins were collected from UniProt through their unique UniProt ID. Enzyme sequences were searched for secretory signal peptide in SignalP-5.0 server.^1^ The tool shows the presence or absence of a secretory signal peptide.

Gene Ontology (GO) enrichment analysis was done using another online tool FungiFun2.^2^ FungiFun2 was used for functional enrichment analysis for fungal genes and proteins. Molecular function and biological process were determined using this tool. A text file was created using UniProt IDs found in LC-ESI-MS/MS of *M. phaseolina*. *M. phaseolina* MS6 strain was selected as the reference species, and GO was selected as classification ontology. Significance level was set at 0.05 (=5.00%). Fisher exact test was selected as significance test, and Benjamini–Hochberg procedure was selected as adjustment method.

### Statistical Analysis

All experiments were done in biological triplicates, and the results were expressed as mean ± standard error, for number of experiments *n* = 3.

## Results

### Isolation and Detection of *Macrophomina phaseolina* Secretome

**FIGURE 1:**
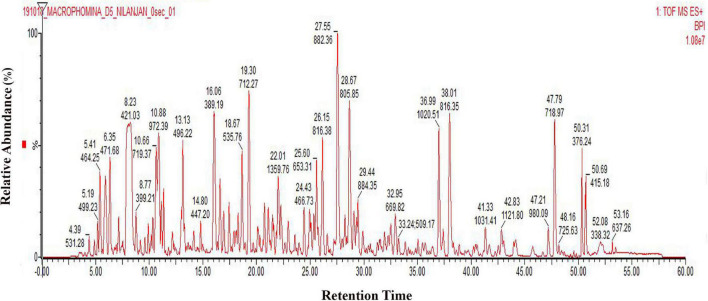
LC-ESI-MS/MS chromatogram of *M. phaseolina* secretome collected 5 days after inoculation. Chromatogram of secretome was generated using 1 mg/mL protein run in Waters-Xevo-G2-X’S-Q-TOF-TOF. The total MS^E^ scan time was 60 min in which 0–5 min was the equilibration time, and then 5–50 min was the elution of peptides from the column, and 50–60 min was the washing of the column.

It has been reported that *M. phaseolina* is seed-borne in nature (Kunwar et al., 1986). It is found both on the seed coat and cotyledons (Reuveni et al., 1983), and it can cause charcoal rot by infecting the roots due to the adherence of microsclerotia to the seed coat during germination (De Mooy and Burke, 1990). Temperature near 30°C and dry conditions make this pathogen prevalent in nature during infection process. Plant pathogenic fungi have the largest proportion of secreted proteins. *M. phaseolina* also infects wheat seeds (Abass et al., 2021). In order to isolate the secretome of *M. phaseolina*, the fungal spore suspension was inoculated in a wheat bran matrix maintaining optimum temperature and relative humidity. This solid substrate culture actually mimics the natural infection process where *M. phaseolina* spores germinate and adhere to wheat bran. *M. phaseolina* secretome was isolated by extracting the wheat bran matrix using phosphate buffer 5, 8, 11, and 14 days after inoculation. Secretome proteins were concentrated using ammonium sulfate precipitation, dialyzed, and run in SDS-PAGE to visualize the secretome profile. Secretome proteins were run three times in SDS-PAGE. Supplementary Figure 1 shows a representative secretome profile of *M. phaseolina* run in SDS-PAGE where the equal amount of proteins was loaded in each lane collected from 5-, 8-, 11-, and 14-day post-inoculated matrix. The secretome showed differences in their protein components with distinctly different protein bands of varied molecular mass visible when compared between the gel lanes charged with samples collected from different postinoculation time periods. The secretome profile of *M. phaseolina* of each lane also varied significantly ranging from low-molecular-weight proteins to high-molecular-weight protein when compared with the molecular weight markers (distributed over a molecular weight range of 180–17 kDa). To identify the whole set of secretory proteins of *M. phaseolina*, the 5-day post-inoculated secretome was selected for LC-ESI-MS/MS analysis because of its low protease secretion in solid substrate culture at that time period. Figure 1 represents the chromatogram of 5-day secretome of *M. phaseolina* run in Waters-Xevo-G2-X’S-Q-TOF-TOF.

### Bioinformatics Analysis of Secreted Proteins

**FIGURE 2:**
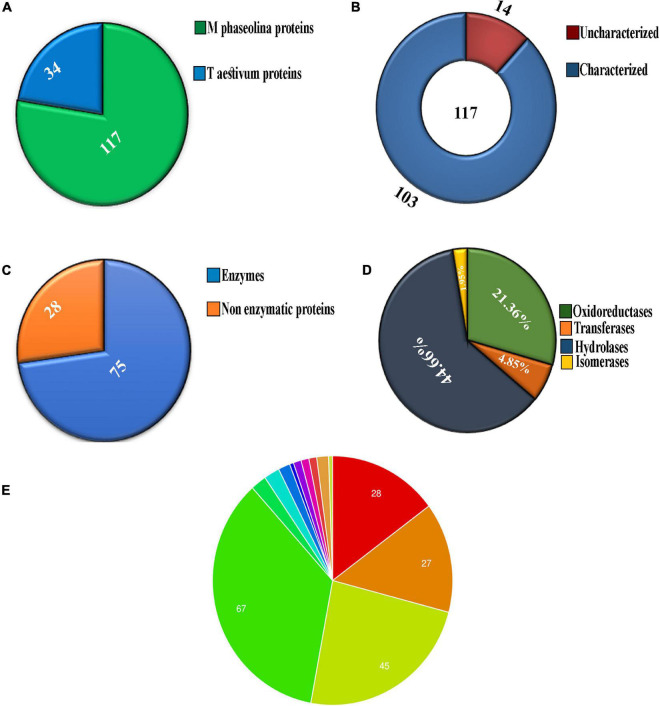
Analysis of secretome obtained from solid-state culture of *M. phaseolina* grown on wheat bran using LC-ESI-MS/MS. Total 151 proteins were found in LC-ESI-MS/MS analysis. **(A)** Pie chart represents the number of *M. phaseolina* and *T. aestivum* proteins found in LC-ESI-MS/MS. Among 151 proteins, 117 proteins were obtained from *M. phaseolina* and 34 proteins from *T. aestivum*. **(B)** Secretome of *M. phaseolina* identified by LC-ESI-MS/MS. Among 117 proteins present in the secretome, 14 proteins were found to be uncharacterized and 103 proteins were found to be characterized. **(C)** Enzymatic and non-enzymatic proteins of *M. phaseolina* identified by LC-ESI-MS/MS. Among 103 characterized proteins, 28 proteins were nonenzymatic, and the rest 75 proteins were enzymes. **(D)** Classification of enzymes found in the secretome of *M. phaseolina*. LC-ESI-MS/MS analysis of the secretome of *M. phaseolina* identified the following four classes of enzymes: (1) isomerases, (2) hydrolases, (3) transferases, and (4) oxidoreductases. Hydrolases were predominantly present in the aforementioned classification. **(E)** Molecular function ontology. GO enrichment analysis was performed to get the molecular functions of genes corresponding to the proteins found in the LC-ESI-MS/MS data of *M. phaseolina*. Most of the genes were found to be involved in different hydrolase activities. Almost 35.5% (67) of the genes corresponding to the proteins are involved in catalytic activity. The involvement of other genes in cellular processes is shown using different color code in the pie chart. All the molecular functions found are as follows: 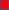 hydrolase activity, hydrolyzing *O*-glycosyl compounds, 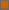 hydrolase activity, acting on glycosyl bonds, 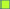 hydrolase activity, 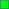 catalytic activity, 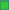 endo-1,4-β-xylanase activity, 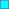 serine-type peptidase activity, 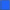 carboxypeptidase activity, 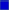 purine ribonucleoside binding, 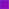 glucosidase activity, 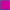 polysaccharide binding, 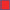 catalase activity, 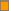 choline dehydrogenase activity, 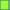 purine ribonucleoside triphosphate binding.

Chromatogram profile indicated the presence of a high percentage of peptide abundance, which would become useful for the identification of secretome proteins of *M. phaseolina.* Whether the chromatogram represents any proteins from wheat bran or not peptide abundance was also searched for *Triticum aestivum* database. LC-ESI-MS/MS data showed that among 151 identified proteins 117 proteins were matched with *M. phaseolina* protein database, and the other 34 proteins were matched with *T. aestivum* protein database (Figure 2A). Supplementary Table 1 represents the list of proteins that matched with *T. aestivum*. It was reported that the *M. phaseolina* genome contains 13.07% (1,863) secreted proteins as compared with 7–10% in other plant pathogens (Islam et al., 2012). Among the 117 identified *M. phaseolina* proteins, 103 proteins were characterized, and 14 proteins were not characterized in the protein database (Figure 2B). Further analysis of the LC-ESI-MS/MS data showed that most of the proteins found in the secretome were enzymes. Among the 103 characterized proteins, 75 proteins were enzymes, and the other 28 proteins were nonenzymatic proteins (Figure 2C). Enzyme classification analysis showed the presence of the following four enzyme classes: (i) isomerases, (ii) hydrolases, (iii) transferases, and (iv) oxidoreductases. Hydrolases are predominantly present (44.66%) in the *M. phaseolina* secretome (Figure 2D).

Gene ontology annotation analysis classified four main molecular functions, which were as follows: (i) hydrolase activity hydrolyzing *O*-glycosyl compounds (14.8%), (ii) hydrolase activity acting on glycosyl bond (14.2%), (iii) hydrolase activity (23.8%), and (iv) catalytic activity (35.5%) (Figure 2E). The other molecular functions include endo-1,4-β-xylanase activity, serine-type peptidase activity, carboxypeptidase activity, purine ribonucleoside binding, glucosidase activity, polysaccharide binding, catalase activity, choline dehydrogenase activity, and purine ribonucleoside triphosphate binding.

### Identification of Secretome Proteins of *Macrophomina phaseolina* by LC-ESI-MS/MS Analysis

It was predicted that *M. phaseolina* contained the lowest number of proteases among ascomycete fungal species (Islam et al., 2012). The list of secretory proteases of *M. phaseolina* found in the LC-ESI-MS/MS is presented in Table 1. Several types of proteases were present, which include peptidase_S9 domain-containing protein, peptidase S8/S53 subtilisin/kexin/sedolisin, peptidase S28, carboxypeptidase, peptide hydrolase, and peptidase M14 carboxypeptidase A. Identification of secretome proteins of *M. phaseolina* was based on their conserved domains and function from the genome sequence.

**TABLE 1:** List of proteases present in the secretome of *M. phaseolina* identified using the LC-ESI-MS-MS.

Accession No.	Peptide abundance	Protein score	Description	Average normalized abundances
K2RI47	17 (17)	222.17	Peptidase_S9 domain-containing protein	1.27e+006
K2R7K1	12 (12)	146.86	Peptidase S8/S53 subtilisin/kexin/ sedolisin	2.56e+006
K2RJW0	8 (8)	94.69	Peptidase S28	6.34e+005
K2RLG4	6 (6)	75.81	Carboxypeptidase	2.74e+005
K2RID0	8 (8)	74.95	Carboxypeptidase	2.73e+005
K2SJI5	4 (4)	65.68	Peptide hydrolase	1.45e+006
K2S0P9	3 (3)	49.7	Peptidase S8/S53 subtilisin/kexin/ sedolisin	1.11e+006
K2R9R2	2 (2)	20.36	Peptide hydrolase	7.61e+004
K2RTD3	2 (2)	19.79	Peptidase M14 carboxypeptidase A	1.14e+005

**FIGURE 3:**
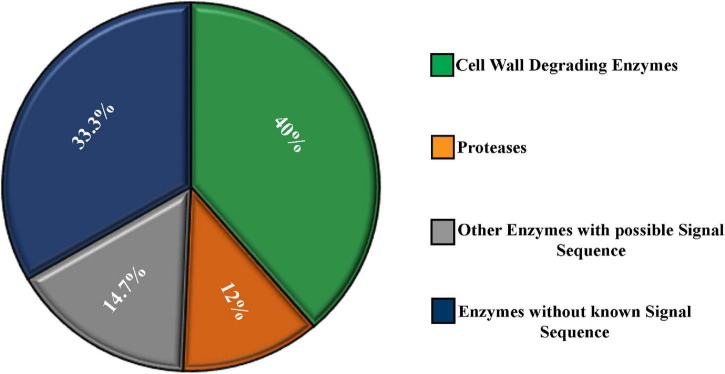
Characterization of the secretome enzymes of *M. phaseolina* identified by LC-ESI-MS/MS analysis. Most of the proteins found in the secretome were enzymes. Almost 40% of the enzymes were cell wall–degrading enzymes. Twelve percent of the enzymes were proteases. A significant portion (14.7%) of the enzymes contains secretory signal sequence, and the rest 33.3% of the enzymes do not contain secretory signal sequence analyzed by signal P.

Almost 40% of the enzymes were cell wall–degrading enzymes. Twelve percent of the enzymes were proteases. The rest, 48%, of enzymes do not contribute in degrading host cell wall but have important functions. A significant portion (14.7%) of these enzymes contained a secretory signal sequence. Approximately 33.3% of the enzymes showed no secretory signal sequence analyzed by signal P (Figure 3).

Phytopathogenic fungi are known to secrete a cocktail of hydrolytic enzymes (including carbohydrate-active enzymes; CAZymes) for degrading the plant cell wall and penetrating into the host tissue (Gibson et al., 2011). *M. phaseolina* genome encodes 362 putative CAZymes including 219 GHs, 56 glycosyltransferases, 65 carbohydrate esterases, 6 carbohydrate binding modules, and 16 polysaccharide lyases comprising more than 80 distinct families (Islam et al., 2012).

### Detection of Cell Wall–Degrading Enzymes Xylanase, Endoglucanase, and Amylase in the Secretome of *Macrophomina phaseolina*

**FIGURE 4:**
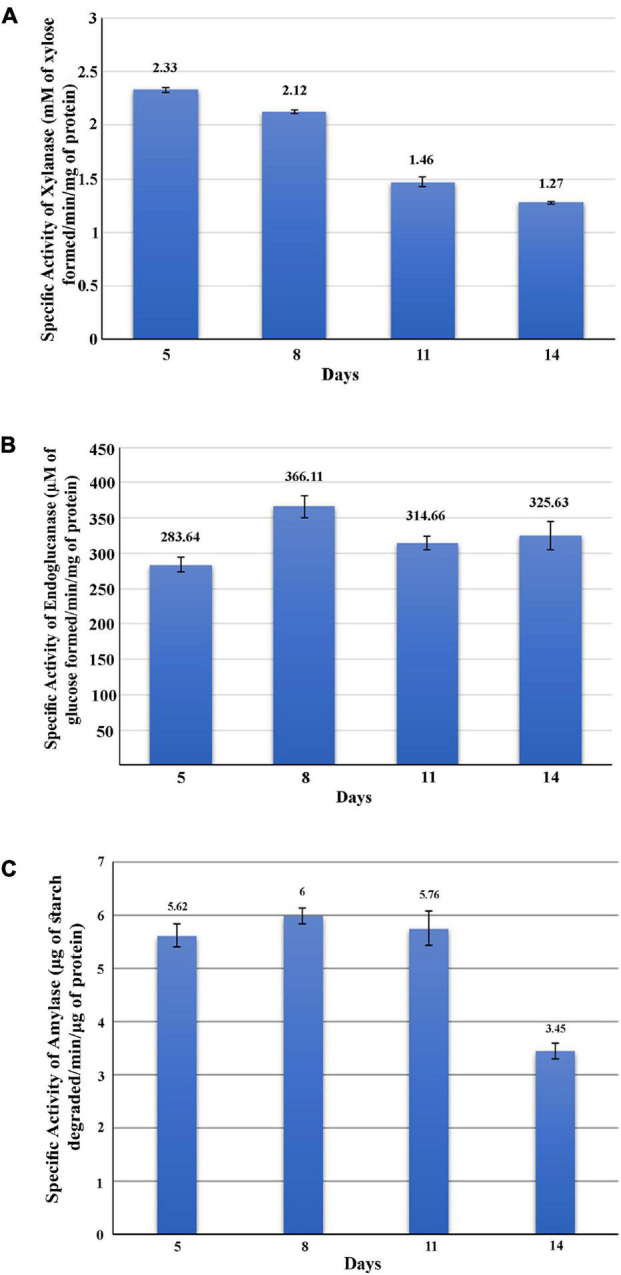
Activity analysis of xylanase, endoglucanase, and amylase enzymes. Secretome of *M. phaseolina* was collected 5, 8, 11, and 14 days after inoculation in solid-state culture, and xylanase, endoglucanase, and amylase activities were determined using spectrophotometric assay. **(A)** Determination of xylanase activity. Specific activity of xylanase was expressed as mM of xylose formed per minute per milligram of secretome protein. **(B)** Determination of endoglucanase activity. Specific activity of endoglucanase was expressed as μM of glucose formed per minute per milligram of secretome protein. **(C)** Determination of amylase activity. Specific activity of amylase was expressed as micrograms of starch degraded per minute per milligram of secretome protein.

**FIGURE 5:**
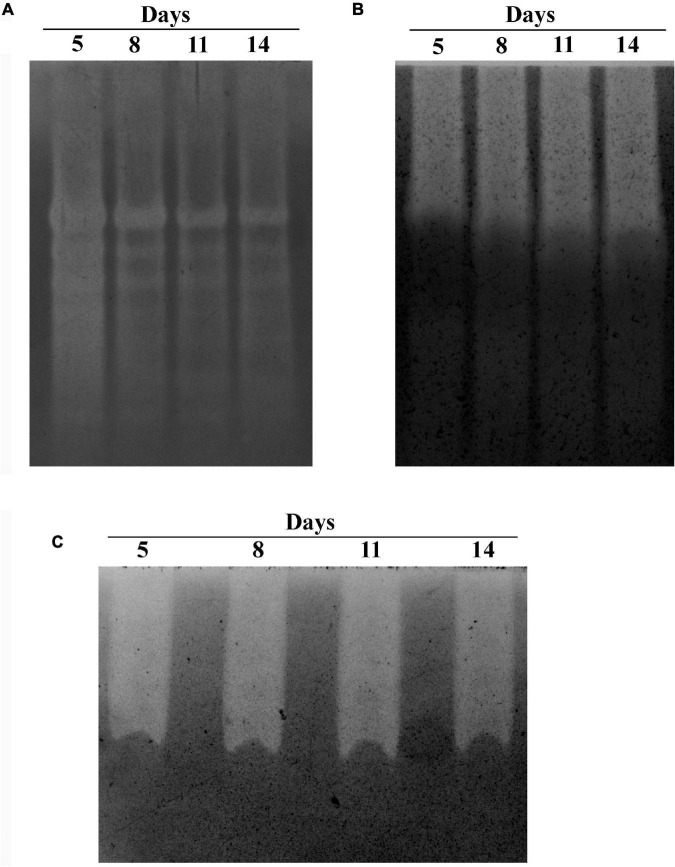
Zymogram analysis of secretory xylanase, endoglucanase, and amylase enzymes. Secretome of *M. phaseolina* was collected 5, 8, 11, and 14 days after inoculation in solid-state culture, and zymogram was studied for xylanase, endoglucanase, and amylase. **(A)** Zymogram analysis of xylanase. The resolving gel was casted with 0.1% birchwood xylan. The gel was stained with 0.1% Congo red and destained with 1 M NaCl solution. The decolorized region in the gel picture indicated the presence of xylanase. **(B)** Zymogram analysis of endoglucanase. The resolving gel was casted with 0.1% CM cellulose. The gel was stained with 0.1% Congo red and destained with 1 M NaCl solution. The decolorized region in the gel picture indicated the presence of endoglucanase. **(C)** Zymogram analysis of amylase. The resolving gel was casted in with 0.1% starch. The gel is stained with iodine solution. The decolorized region in the gel picture indicated the presence of amylase.

**FIGURE 6:**
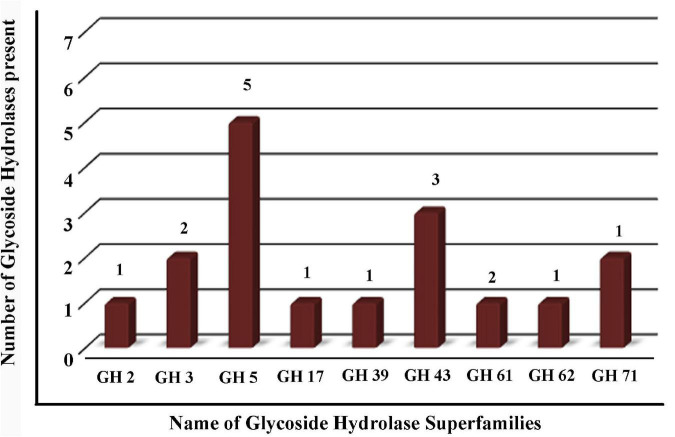
Profile of glycoside hydrolases (GHs) found in the secretome proteins of *M. phaseolina* identified by LC-ESI-MS/MS. Bar graph shows the distribution of 17 GHs identified by LC-ESI-MS/MS. Nine different types of GHs were found in multiple numbers in the secretome proteins of *M. phaseolina*.

The GHs family was highly represented in the *M. phaseolina* secretome. Specific activity of xylanase was detected in the *M. phaseolina* secretome at 5-, 8-, 11-, and 14-day postinoculation time period (Figure 4A). The highest xylanase-specific activity (2.33 ± 0.000026 mM of xylose formed per minute per milligram of protein) was found at 5-day postinoculation time period. It was gradually decreased at 8-, 11-, and 14-day postinoculation time period. Xylanase activity was further detected in zymogram (Figure 5A). Zymogram profile of xylanase activity present in the secretome of *M. phaseolina* was corroborated well with the specific activity determination. Endoglucanase-specific activity of *M. phaseolina* secretome was highest (366.11 ± 16.1 μM of glucose formed per minute per milligram of protein) at 8-day postinoculation time period (Figure 4B). There was no significant change in endoglucanase-specific activity of *M. phaseolina* secretome in the 11- and 14-day postinoculation time period compared with the 8-day postinoculation time period. Similar trend was observed in zymogram profile of endoglucanase activity present in the secretome of *M. phaseolina* (Figure 5B). Amylase-specific activity of *M. phaseolina* secretome was the highest (6 ± 0.15 μg of starch degraded per minute per microgram of protein) at the 8-day postinoculation time period (Figure 4C). There was a significant decrease in the amylase-specific activity of *M. phaseolina* secretome at the 14-day time period. Zymogram profile of amylase activity also supported the trend observed in specific activity determination (Figure 5C). Nine families of GHs present in the *M. phaseolina* secretome were found to be involved in the degradation of carbohydrate complexes. Figure 6 represents the profile of GHs found in the secretome proteins of *M. phaseolina* identified by LC-ESI-MS/MS.

### Identification of Secretome Proteins in the LC-ESI-MS/MS Based on the Presence or Absence of Secretory Signal Sequence

**FIGURE 7:**
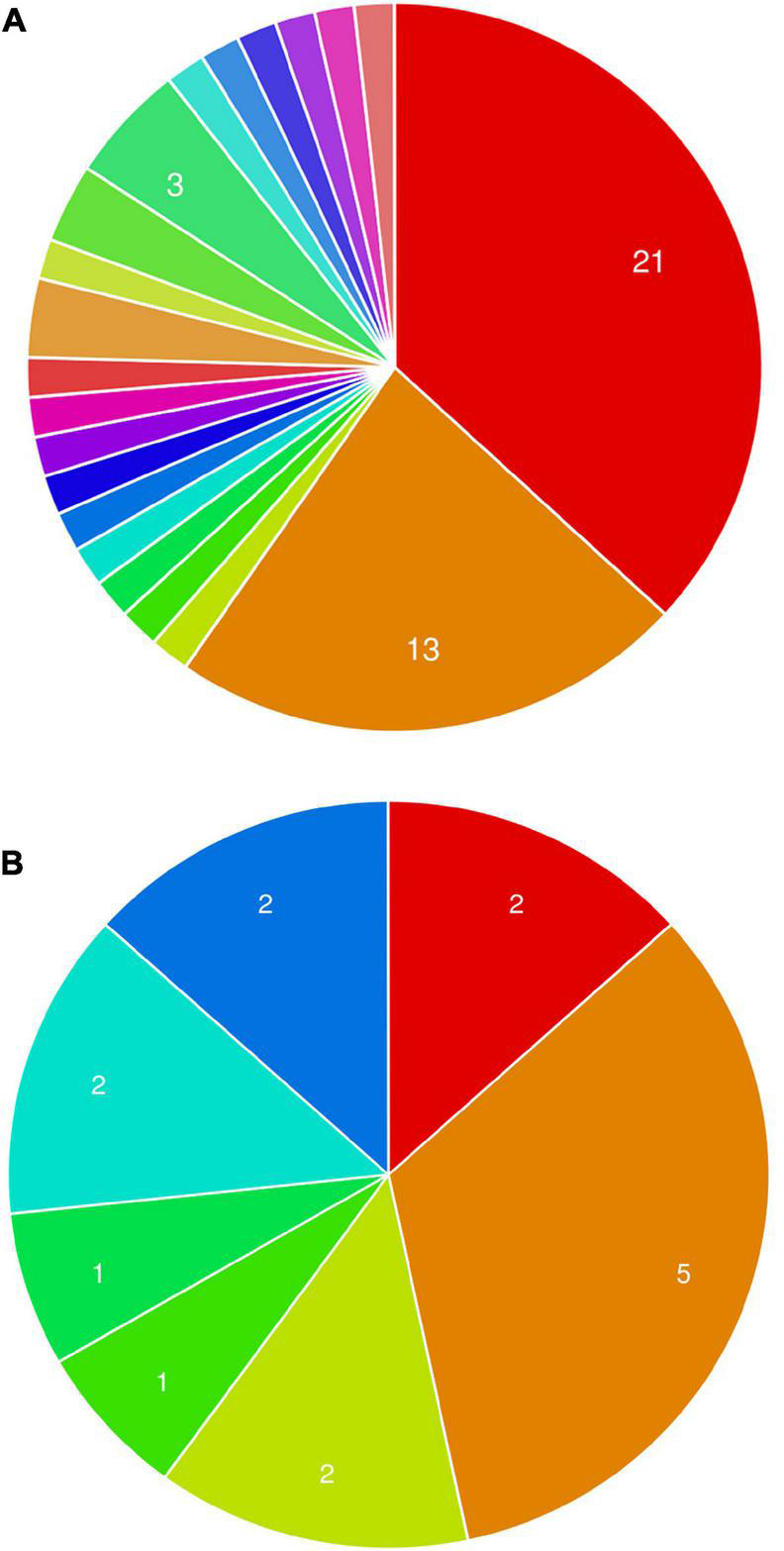
Molecular function ontology of enzymes analyzed by signal P found in LC-ESI-MS/MS of *M. phaseolina* secretome. The enzymes other than the cell wall–degrading enzymes and proteases found in LC-ESI-MS/MS were first categorized based on the presence of the secretory signal peptide by signal P online tool. GO enrichment analysis was done using the UniProt IDs of the enzymes. **(A)** Molecular function of the genes corresponding to the enzymes that contain a signal sequence found in the LC-ESI-MS/MS. Almost 37.5% of the genes corresponding to the enzymes are involved in hydrolase activity. All the molecular functions found are as follows: 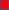 catalytic activity, 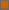 oxidoreductase activity, 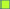 inorganic diphosphatase activity, 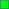 nucleoside diphosphate kinase activity, 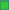 glutathione transferase activity, 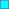 citrate (Si)-synthase activity, 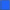 L-malate dehydrogenase activity, 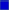 sedoheptulose-7-phosphate:D-glycreraldehyde-3-phosphate glyceronetransferase activity, 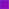 phosphatidylininositol-3-phosphate binding, 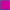 triose-phosphate isomerase activity, 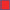 phosphatidylinositol phosphate binding, 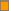 antioxidant activity, 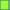 homogentisate 1,2-dioxygenase activity, 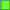 isomerase activity, 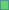 oxidoreductase activity, acting on CH-OH group of donors, 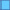 malate dehydrogenase activity, 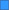 oxidoreductase activity, acting on superoxide radicals as acceptor, 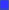 superoxide dismutase activity, 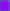 transferase activity, transferring aldehyde or ketonic groups, 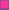 pyrophosphatase activity, 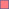 nucleobase-containing compound kinase activity. **(B)** Molecular function of the genes corresponding to the enzymes that do not contain a signal sequence found in the LC-ESI-MS/MS. Almost 36.9% of the genes corresponding to the enzymes are involved in catalytic activity, and 22.8% of the genes corresponding to the enzymes are involved in oxidoreductase activity. All the molecular functions found are as follows: 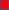 choline dehydrogenase activity, 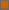 hydrolase activity, 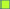 hydrolase activity, acting on carbon-nitrogen (but not peptide) bonds, 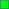 catalase activity, 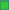 hydrolase activity, acting on carbon-nitrogen (but not peptide) bonds, in linear amidines, 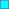 flavin adenine dinucleotide binding, 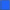 oxidoreductase activity, acting on CH-OH group of donors.

Table 2 represents list of enzymes without having known signal sequence found in the LC-ESI-MS/MS protein identification. Table 3 represents the list of enzymes with signal sequence as identified by signal P found in the LC-ESI-MS/MS data of the secretome proteins of *M. phaseolina*. Figures 7A,B show the molecular function ontology of the genes corresponding to their proteins presented in Tables 2, 3. Most of the proteins showed catalytic activity, and they belong to the group of virulence factors. The number of genes involved in the molecular function has been shown within the color code. A diverse group of enzymes was found in the secretome starting from metabolic enzyme, redox active enzymes, to genotoxic compound detoxifying enzymes. Proteins that provide protection against reactive oxygen species (ROS)–based plant defenses may be responsible for stronger pathogenicity of *M. phaseolina*. ROS generation is the earliest response of plant toward the invading pathogen. Production of ROS alters the host cells’ redox status and activates various plant defense responses, including production of pathogenesis related proteins, phytoalexins, and signaling molecules (Huang et al., 2019). This creates an inhospitable situation for the pathogens, and thus pathogens must degrade or neutralize such ROS in order to overcome the host defense responses. A recent study published from our laboratory showed the presence of catalase, catalase peroxidase, and superoxide dismutase in *M. phaseolina* secretome. In that study, proteins such as superoxide dismutase [Cu-Zn] and catalase–peroxidase were found without having known signal sequence. We also identified a catalase protein with signal sequence as identified by signal P found in the LC–ESI-MS/MS data of the secretome proteins of *M. phaseolina* (Sinha et al., 2021). These proteins play an essential role in protecting the pathogen against the host-derived ROS and therefore overcoming the host defenses and creating a successful infection. Antioxidant proteins such as thioredoxins and glutathione S-transferase were also identified in the secretome of *M. phaseolina*. Identified enzymes such as glucose–methanol–choline (GMC) oxidoreductase, aldo/keto reductase (AKR), formate dehydrogenase, alcohol dehydrogenase (ADH) superfamily zinc-containing, malate dehydrogenase, peptidyl-prolyl-*cis*-*trans* isomerase, AKR, homogentisate 1 2-dioxygenase, lipase GDSL, inorganic pyrophosphatase, nucleoside diphosphate kinase (NDK), citrate synthase, transaldolase, triosephosphate isomerase, short-chain dehydrogenase/reductase, peptidylprolyl isomerase, dienelactone hydrolase, and epoxide hydrolase are also known virulence factors present in many plant pathogenic fungi.

**TABLE 2:** List of enzymes without having known signal sequence as identified by Signal P found in the LC-ESI-MS-MS of *M. phaseolina* secretome.

Accession No.	Peptide abundance	Protein Score	Description	Average normalized abundances
K2R576	25 (25)	319.35	Glucose-methanol-choline oxidoreductase	2.36e+006
K2RDL9	18 (18)	251.32	Aldo/keto reductase	2.40e+006
K2SX20	16 (16)	207.83	Formate dehydrogenase	1.43e+006
K2R950	16 (16)	189.04	Alcohol dehydrogenase superfamily zinc-containing	1.43e+006
K2SB76	14 (12)	164.65	Malate dehydrogenase	8.63e+005
K2S4D8	11 (11)	128.83	Aldo/keto reductase	3.18e+005
K2RWZ0	9 (9)	110.59	Superoxide dismutase [Cu-Zn]	1.89e+006
K2QZ33	13 (13)	105.65	Catalase-peroxidase	8.12e+005
K2SS52	10 (10)	100.04	Homogentisate 1 2-dioxygenase	4.46e+005
K2R7M0	7 (7)	89.46	Lipase GDSL	7.51e+005
K2RV72	7 (7)	89.12	Alcohol dehydrogenase superfamily zinc-containing	2.56e+005
K2REA4	5 (5)	71.92	Peptidyl-prolyl cis-trans isomerase	8.19e+005
K2SJD8	6 (6)	63.24	Glutathione S-transferase	2.81e+005
K2RD42	6 (6)	60.55	Alcohol dehydrogenase superfamily zinc-containing	1.20e+005
K2SD06	6 (6)	60.35	Transaldolase	2.82e+005
K2RAK3	6 (6)	56.9	Aldo/keto reductase	2.25e+005
K2S262	6 (6)	56.47	Glutathione S-transferase	3.12e+005
K2RI19	6 (6)	55.2	Inorganic pyrophosphatase	1.71e+005
K2S9J1	4 (4)	48.54	Nucleoside diphosphate kinase	3.57e+005
K2SJ45	4 (4)	40.82	Triosephosphate isomerase	1.24e+005
K2S1F3	5 (5)	37.3	Short-chain dehydrogenase/ reductase SDR	2.50e+005
K2REF5	5 (5)	33.76	Citrate synthase	9.86e+004
K2RH98	2 (2)	21.74	Epoxide hydrolase	9.57e+004
K2RF71	2 (2)	20.13	Dienelactone hydrolase SV = 1	2.04e+005
K2QU25	3 (3)	19.81	Aldo/keto reductase	5.09e+004

**TABLE 3:** List of enzymes with signal sequence as identified by Signal P found in the LC–ESI-MS-MS data of *M. phaseolina* secretome.

Accession No.	Peptide abundance	Protein score	Description	Average normalized abundances
K2RRJ6	18 (18)	235.88	Glucose-methanol-choline oxidoreductase	2.22e+006
K2QYR6	10 (10)	160.41	Esterase PHB depolymerase	8.00e+006
K2RBI3	9 (9)	157.06	Lipase GDSL	6.69e+006
K2RGV8	9 (9)	130.47	Ureohydrolase	1.67e+006
K2RBR1	8 (8)	119.53	Neuraminidase	1.10e+006
K2R4N1	9 (9)	85.16	Catalase	4.13e+005
K2RE08	9 (9)	83.54	Glucose-methanol-choline oxidoreductase	2.77e+005
K2RK28	6 (6)	83.26	Lipase class 3	1.71e+006
K2RAG2	6 (6)	82.15	Polysaccharide deacetylase	5.43e+005
K2RQT3	5 (5)	77.64	Lipase GDSL	9.10e+005
K2SSW0	4 (4)	31.42	Neuraminidase	1.48e+005

Plant cell wall–degrading enzymes require the assistance of carbohydrate esterases (Aspeborg et al., 2012) to deacetylate the substituted saccharides (esters or amides) of celluloses (Biely, 2012). Our LC-ESI-MS/MS data showed the presence of polysaccharide deacetylase. Secretome data showed the presence of the GHs, which include choline dehydrogenase activity, hydrolase activity, β-galactosidase activity, catalase activity, hydrolase activity acting on carbon–nitrogen bond but not on peptide bonds, and oxidoreductase activity acting on CH-OH group of donors (Figure 7B).

## Discussion

Necrotrophic fungal pathogens have evolved various mechanisms to kill their host quickly to feed themselves and complete their lifecycle. They are generally resistant to a hypersensitive response, indicating that they possess a repertoire of effectors to counteract host-generated oxidative stress and to induce host cell death. Among the necrotrophic fungal pathogens, *M. phaseolina* is one important soil-borne necrotrophic pathogen that infects more than 500 plant species around the world (Wyllie, 1998). Thus, it is very important to identify the *M. phaseolina* secretome, which is used as tool box to destroy the host plant. The term *fungal secretome* now refers to a set of secretory proteins and enzymes with or without signal peptide, proteins from exosomes (Meijer et al., 2014; Vincent et al., 2019). Studies on the whole-genome sequence of *M. phaseolina* predicted that this phytopathogen has an abundance of secreted oxidases, peroxidases, and hydrolytic enzymes for degrading cell wall polysaccharides and lignocelluloses to penetrate into the host tissue (Islam et al., 2012). For the identification of *M. phaseolina* secretome, we developed a successful and reproducible bioprocess in this study. By virtue of its secreted enzymes, *M. phaseolina* are potent decomposers of plant cell walls. Plant cell walls are composed mainly of cellulose, lignin, and hemicellulose. This composite is often referred to as lignocelluloses. As the cell wall imparts a significant barrier to pathogen entrance, necrotrophs, especially *M. phaseolina*, have evolved mechanisms to overcome this barrier. Our secretome analysis showed that *M. phaseolina* secretes numerous cell wall–degrading enzymes to disrupt the cell and for absorptive nutrition. Similar observation was found in necrotrophic fungi *Botrytis cinerea*, which penetrate the host cell wall by releasing the cell wall–degrading enzymes rather than by physical force (Choquer et al., 2007). GHs are the most diverse group of enzymes used by microbes in the degradation of lignocelluloses (Murphy et al., 2011). It is evident from our study that *M. phaseolina* also relies on producing different family of GH when it was grown in wheat bran matrix. Plant cell wall–degrading enzymes require the assistance of carbohydrate esterases (Aspeborg et al., 2012) to deacetylate the substituted saccharides (esters or amides) of celluloses (Yagminas et al., 1990). Our LC-ESI-MS/MS data showed the presence of polysaccharide deacetylase. β-Glucosidase has been identified as a virulence factor in other microorganisms: it is directly involved in the virulence of the phytopathogenic *Pyrenophora tritici-repentis* (anamorph: *Drechslera tritici-repentis*), the necrotrophic fungus responsible for tan spot (Fu et al., 2013). The presence of β-glucosidase in *M. phaseolina* secretome is in agreement with the previously published reports by Reuveni et al. (2007). Therefore, GHs appeared to be the putative virulence factors for *M. phaseolina*. A short-chain dehydrogenase/reductase was identified in *M. phaseolina* secretome grown in wheat bran matrix. It has been reported that a short-chain dehydrogenase/reductase gene is required for infection-related development and pathogenicity in *Magnaporthe oryzae*, a phytopathogenic fungus (Kwon et al., 2010).

In this study, we identified a list of proteases in solid-state culture of *M. phaseolina*. During encounter with the host plant, pathogenic fungi produce various cell wall–degrading enzymes and proteases to fragment the plant cell wall polymers and thereby facilitate penetration into the host cells (Doi and Kosugi, 2004; Kubicek et al., 2014; Jashni et al., 2015; Quoc and Chau, 2017). Thus, these *M. phaseolina* proteases may have certain role in cell wall degradation and as putative virulence factors during plant–pathogen interactions.

We identified neuraminidase in the *M. phaseolina* secretome collected from wheat bran matrix. Neuraminidase is an exoglycosidase that cleaves glycoconjugates, releasing the terminal sialic acid residues (Warwas et al., 2010). The identification of neuraminidase in *M. phaseolina* secretome corroborated well with the previous finding where it was shown that *M. phaseolina* recognizes and infects host plants *via* a sialic acid–binding lectin (Bhowal et al., 2005, 2006). These enzymes are involved in the virulence of several microorganisms (Jermyn and Boyd, 2002; Zhou et al., 2009; Warwas et al., 2010).

In the present study, GMC oxidoreductase was identified in the *M. phaseolina* secretome collected from wheat bran matrix. GMC oxidoreductases are flavoenzymes that catalyze the electron transfer between molecules by utilizing diverse substrates and are exclusively found in members of Basidiomycota and Pezizomycotina. In phytopathogenic fungi, these enzymes play important role in various processes, including browning, pigmentation, pathogen defense, virulence mechanisms, and melanin production. The identified GMC oxidoreductase in *M. phaseolina* secretome may play a dual role. First, they may defend the fungal pathogens from oxidative stress by acting as scavenger of ROS generated by the host plant. Second, they may prevent the activation of host defense mechanisms downstream to ROS production as found in *Tilletia indica*, which is smut fungus that causes Karnal bunt (KB) disease in wheat (Pandey et al., 2018). AKR is found to play an adaptive role in another necrotrophic pathogen *Beauveria bassiana* (Wang et al., 2018).

In this study, thioredoxin was identified in *M. phaseolina* secretome collected from wheat bran matrix. Thioredoxins are small disulfide containing redox proteins. They are protein disulfide oxidoreductases that regulate the cellular redox status and defend the fungal pathogen against ROS accumulation (Li et al., 2016). Thioredoxins are highly conserved among living organisms. In chestnut blight pathogen, corn smut fungus *Ustilago maydis* is found to secrete a pathogenicity-related thioredoxin (Muller et al., 2008). In *Alternaria brassicicola*, which are the causative agent of brassica dark leaf spot, thioredoxin gene expression has been detected following exposure to defense metabolites (Kang et al., 2020). That is why thioredoxin has been classified as stress, oxidative burst, and defense gene in causal agent of brassica dark leaf spot.

Triose phosphate isomerase was identified in the secretome of *M. phaseolina* grown in wheat bran matrix. It catalyzes the reversible conversion of dihydroxyacetone phosphate and D-glyceraldehyde 3-phosphate in glycolysis. In pathogenic fungus *Paracoccidioides brasiliensis*, triose phosphate isomerase has been found to play a crucial role in pathogen adherence to host and involved in host cell invasion (Pereira et al., 2007). It was also found in highly virulent *T. indica* fungus that causes KB disease in wheat (Pandey et al., 2018). Triose phosphate isomerase may play similar function in *M. phaseolina* during pathogenesis.

Plant pathogenic fungi secrete several lipases that hydrolyze the carboxyl ester bonds from fatty acid polymers. Fungal lipases are known to be involved in fungal penetration through the plant barriers such as cuticle and waxes (Serrano et al., 2014). The potential role of secreted lipase as fungal virulence factor has been identified first in necrotrophic fungus *B. cinerea*, a causal agent of gray mold disease of grapes (Reis et al., 2005). In *U. maydis*, secreted lipase activity has been found to promote the transition of the fungus to pathogenic filamentous state (Klose et al., 2004). We identified the presence of secreted lipase in the *M. phaseolina* secretome, which was grown in wheat bran matrix. Thus, it seems that secreted lipases may play a crucial role in host–pathogen interactions and are involved in fungal pathogenesis. Esterase PHB depolymerase is involved in lipid degradation. It is involved in the hydrolysis of carboxylic ester. PHB depolymerase was identified in the secretome of *M. phaseolina* grown in wheat bran matrix. It has also been reported in the predicted protein secretome of the fungal wheat leaf pathogen *Mycosphaerella graminicola* (Morais do Amaral et al., 2012).

Feruloyl esterases (FAEs, EC 3.1.1.73) are auxiliary enzymes in the degradation of lignocellulosic complexes of the cell wall, through the hydrolysis of the ester bonds that join hydroxycinnamic acids (e.g., ferulic acid) and lignin–carbohydrate complexes. We identified feruloyl esterase in the secretome of *M. phaseolina* grown in wheat bran matrix. Feruloyl esterase has been identified in the secretome of *Alternaria alternata*, which is capable of damaging a wide spectrum of plant hosts (Garcia-Calvo et al., 2018). Epoxide hydrolase was also identified in secretome of *M. phaseolina* grown in wheat bran matrix. The function of epoxide hydrolase (EC 3.3.2.3) is to catalyze the hydrolysis of epoxides or arene oxides to their corresponding diols by the addition of water (Pinot et al., 1997). Little is known about epoxide hydrolases from filamentous fungi. An epoxide hydrolase was found to be induced by *Fusarium solani pisi* by a cutin extract of plants (Kolattukudy and Brown, 1975). This enzyme is apparently related to the ability of the mycelium to infect some plants (Morisseau et al., 1999). The AKRs are a superfamily of enzymes with diverse functions in the reduction of aldehydes and ketones. AKR was identified in the secretome of *M. phaseolina* grown in wheat bran matrix. This enzyme has been reported in *B. bassiana*, a necrotrophic fungus that has evolved the ability to infect and parasitize insect, which is also a saprophyte found in soil, and can form endophytic relationships with various plants (Behie and Bidochka, 2014). *M. phaseolina* secretome was found to contain formaldehyde dehydrogenase. It has been reported that formaldehyde dehydrogenase was also expressed in *Sclerotinia sclerotiorum* Fdh1 in fungal pathogenesis (Zhu et al., 2019). ADHs are a group of oxidoreductases that occur in many organisms facilitating the conversion between alcohols and aldehydes with the reduction of NAD^+^. Alcohol dehydrogenase gene (ADH1) disruption in plant pathogenic fungus *Fusarium oxysporum* impaired growth under hypoxic conditions, diminished production of ethanol, and affected the fungal disease development in tomato plants (Corrales Escobosa et al., 2011). ADH could be induced by hypoxic condition and influence fungal growth and pathogenesis in *Aspergillus fumigatus* (Grahl et al., 2011). Interestingly, ADH was also identified in *M. phaseolina* secretome. ADHs may play a similar role in *M. phaseolina* during growth and pathogenesis. We identified malate dehydrogenase enzyme in the secretome of *M. phaseolina.* Malate dehydrogenase enzyme catalyzes the reversible conversion of oxalacetate and malate. In *B. cinerea*, oxalacetate has been described as a pathogenicity factor (Lyon et al., 2004), as the secretion of oxaloacetic acid may create an acidic environment that facilitates the pathogenic activity of the fungus. Homogentisate 1 2-dioxygenase was also identified in *M. phaseolina* secretome. Homogentisate 1,2-dioxygenase catalyzes the conversion of homogenistic acid into maleylacetoacetic acid in the tyrosine metabolism pathway. Tyrosine is assimilated *via* a conserved catabolic pathway that provides the fungus with both nitrogen, from a transamination reaction with α-ketoglutarate to produce glutamate, and carbon *via* production of fumarate and acetoacetate, which is used to generate acetyl-CoA that can then feed into the TCA cycle. Tyrosine is an important precursor in the formation of two different types of melanin: DOPA melanin *via* the metabolism of tyrosine to form DOPA and pyomelanin through the oxidation and polymerization of homogentisate during tyrosine catabolism (Fernandez-Canon and Penalva, 1995; Schmaler-Ripcke et al., 2009). Pyomelanin has been reported to protect *Penicillium marneffei* against oxidative stress. The fundamental biological function of NDK is to catalyze the reversible exchange of the -phosphate between nucleoside triphosphate (NTP) and nucleoside diphosphate (NDP). NDK was identified in *M. phaseolina* secretome collected from wheat bran matrix. NDK family proteins are ubiquitous enzymes that exchange the -phosphate between NTP and NDP, preserving NTPs in the cell. NDK has been known to play an important role in spore development, sclerotia production, and pathogenicity on seeds In *Aspergillus flavus*. Similar function of this enzyme can be expected in *M. phaseolina*. The usefulness of the present experimental setup is to get high-yielding secretory proteins from *M. phaseolina* when it was grown in wheat bran matrix. Moreover, downstream processing of the secretome requires less purification steps. It has also been reported that *M. phaseolina* also infects wheat seeds (Abass et al., 2021). Thus, the putative virulence factors that were identified in the present study may help to deduce the infection mechanisms involved in other susceptible host plants. It is very difficult to get the pure fungal secretome from the mixed system of the infected plant tissues. That is why apoplasts have been used in secretome analysis in a few cases. However, solid-state culture for *M. phaseolina* can provide similar pathological settings when seed cotyledons are infected. Although few wheat proteins have been identified in this secretome, that is small in numbers in the context of whole secretome.

## Conclusion

Although during the last five decades, extensive work has been made by the researchers in the areas of etiology, epidemiology, biology, and biocontrol of the ascomycete fungus *M. phaseolina*, still control of charcoal rot diseases is difficult. Based on the whole-genome sequence, it is possible to examine secretome using analytical and computational tools. For predicted proteins, the accuracy of a signal peptide prediction heavily relies on an accurate gene model, which provides a correct N-terminal end sequence of the encoded protein. However, some proteins will not actually be secreted, even with a correctly predicted signal peptide, for instance, because they are resident endoplasmic reticulum proteins. On the other hand, the presence of intracellular proteins might be explained by exosomes and microvesicles, which actually add complexity to secretome studies. That is why the proteomic analysis of secretome of pathogens may identify the actual process or the involvement of proteins related to pathogenicity or putative virulence factors. ROS seems to be induced following *M. phaseolina* infection in seed coats of wheat as several proteins involved in oxidative stress defense such as superoxide dismutase, catalase, and thioredoxins were identified in the secretome analysis.

## Data Availability Statement

The data presented in the study are deposited in the ProteomeXchange repository, accession number PXD032749.

## Author Contributions

NS and SP performed the experiments and analyzed data. NS and SG wrote the manuscript with the contribution of SP. All authors conceived the research and approved the submitted version.

## Conflict of Interest

The authors declare that the research was conducted in the absence of any commercial or financial relationships that could be construed as a potential conflict of interest.

## Publisher’s Note

All claims expressed in this article are solely those of the authors and do not necessarily represent those of their affiliated organizations, or those of the publisher, the editors and the reviewers. Any product that may be evaluated in this article, or claim that may be made by its manufacturer, is not guaranteed or endorsed by the publisher.

## Footnotes

1
http://www.cbs.dtu.dk/services/SignalP/

2
https://elbe.hki-jena.de/fungifun/

## Supplementary Material

Supplementary Figure 1 Secretome profile of *M. phaseolina* grown in solid state culture using wheat bran as substrate. Secretome of *M. phaseolina* was collected after 5, 8, 11 and 14 days post inoculation in solid state culture, and 50 μg extracted protein was run in SDS-PAGE. Molecular weight markers were also run in the same gel.
