# Supplementary figures and images for: Secretome Analysis of Macrophomina phaseolina Identifies an Array of Putative Virulence Factors Responsible for Charcoal Rot Disease in Plants
Source: Front Microbiol. 2022 Apr 5;13:847832. doi: 10.3389/fmicb.2022.847832 (PMC9037145; doi:10.3389/fmicb.2022.847832)

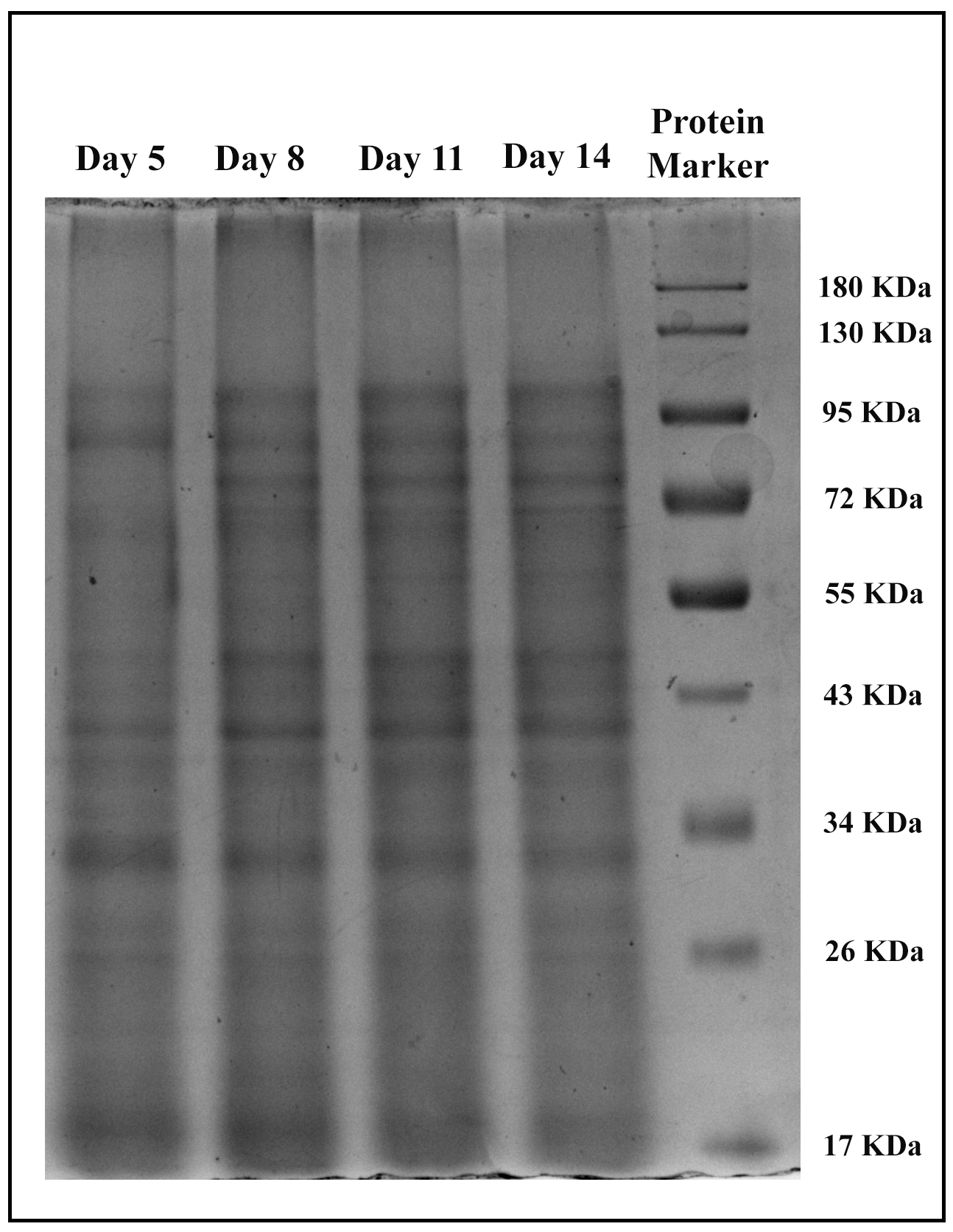

Supplement: Supplementary Figure 1 — Secretome profile of M. phaseolina grown in solid state culture using wheat bran as substrate. Secretome of M. phaseolina was collected after 5, 8, 11 and 14 days post inoculation in solid state culture, and 50 μg extracted protein was run in SDS-PAGE. Molecular weight markers were also run in the same gel. [file Image_1.TIF]
