# Supplementary material for: Secretome Analysis of Macrophomina phaseolina Identifies an Array of Putative Virulence Factors Responsible for Charcoal Rot Disease in Plants
Source: Front Microbiol. 2022 Apr 5;13:847832. doi: 10.3389/fmicb.2022.847832 (PMC9037145; doi:10.3389/fmicb.2022.847832)
Supplement: Supplementary file 2 [file Table_1.doc]

**Table S1: List of proteins matched with the database of *Triticum aestivum* in the LC-ESI-MS-MS**

| **Accesion No.** | **Peptide abundance** | **Protein Score** | **Description** | **Average Normalised Abundances** |
| --- | --- | --- | --- | --- |
| I6QQ39 | 17 (5) | 183.16 | Globulin-3A | 1.20e+006 |
| A0A3B6IJ76 | 13 (0) | 157.42 | Uncharacterized protein | 1.04e+006 |
| A0A3B6ILV9 | 11 (0) | 154.37 | Uncharacterized protein | 1.07e+006 |
| A0A3B6JCJ8 | 11 (2) | 152.24 | Uncharacterized protein | 1.05e+006 |
| B7U6L5 | 9 (0) | 121.79 | Globulin 3B | 1.02e+006 |
| Q7DMU0 | 10 (1) | 118.54 | Storage protein | 7.86e+005 |
| A0A3B6JBZ2 | 9 (1) | 113.56 | Uncharacterized protein | 1.01e+006 |
| A0A3B6TZD9 | 6 (2) | 83.4 | Chitinase | 4.14e+005 |
| A0A3B6RP58 | 5 (1) | 67.15 | Chitinase | 3.80e+005 |
| Q4Z8L8 | 7 (5) | 58.12 | Chitinase | 3.13e+005 |
| Q8L5C6 | 5 (2) | 55.08 | Xylanase inhibitor protein 1 | 7.44e+005 |
| Q4W6G2 | 4 (1) | 53.27 | Xylanase inhibitor XIP-III | 2.10e+005 |
| A0A3B6B2R3 | 4 (4) | 34.35 | Uncharacterized protein | 8.32e+004 |
| A0A3B5Z5T0 | 3 (1) | 34.26 | Malate dehydrogenase | 1.34e+005 |
| A0A3B6TAH3 | 2 (2) | 28.35 | Uncharacterized protein | 3.11e+005 |
| A0A077RTG8 | 3 (1) | 25.25 | Malate dehydrogenase | 7.21e+004 |
| R4ZAP0 | 3 (3) | 24.53 | L-2 | 1.56e+005 |
| A0A3B5XTN8 | 2 (1) | 23.12 | Uncharacterized protein | 1.45e+005 |
| A0A3B6JK40 | 3 (1) | 21.5 | Ubiquitin | 6.53e+004 |
| Q8GZB0 | 3 (3) | 19.26 | Non-specific lipid-transfer protein | 1.15e+005 |
| O64393 | 1 (1) | 16.67 | Wheatwin-2 | 2.80e+005 |
| A0A080YUA6 | 1 (1) | 15.28 | Barwin domain-containing protein | 7.84e+004 |
| G9DR81 | 2 (2) | 13.7 | Genome assembly_ chromosome: II | 9.25e+004 |
| A0A3B6QCG8 | 2 (2) | 11.38 | LRRNT_2 domain-containing protein | 3.57e+004 |
| A0A077RWU9 | 2 (2) | 11.15 | ULP_PROTEASE domain-containing protein | 5970.08 |
| A0A3B6KUN7 | 1 (1) | 7.1 | AAI domain-containing protein | 5531.39 |
| A0A3B5Z038 | 1 (1) | 6.98 | BZIP domain-containing protein | 9.93e+004 |
| A0A3B6ML31 | 1 (1) | 6.21 | TF-B3 domain-containing protein | 1.66e+004 |
| A0A077RQM5 | 1 (1) | 5.88 | ANK_REP_REGION domain-containing protein | 2317.99 |
| A0A3B6K9Y8 | 1 (0) | 5.86 | Lactamase_B domain-containing protein | 3.24e+005 |
| A0A3B6FIS3 | 1 (1) | 5.8 | Lysine--tRNA ligase | 2529.26 |
| A0A077RWN8 | 1 (1) | 5.73 | Uncharacterized protein | 2818.68 |
| A0A3B6BWS0 | 1 (1) | 5.16 | Terpene_synth domain-containing protein | 7818.25 |
| A0A3B6TN54 | 1 (0) | 4.8 | Uncharacterized protein | 7.74e+004 |
